# The relationship between synovial inflammation, structural pathology, and pain in post-traumatic osteoarthritis: differential effect of stem cell and hyaluronan treatment

**DOI:** 10.1186/s13075-020-2117-2

**Published:** 2020-02-14

**Authors:** Cindy C. Shu, Sanaa Zaki, Varshini Ravi, Antonella Schiavinato, Margaret M. Smith, Christopher B. Little

**Affiliations:** 1 Raymond Purves Bone and Joint Laboratory, Institute of Bone and Joint Research, Kolling Institute, Faculty of Medicine and Health, University of Sydney, Level 10 Kolling Building – B6, Royal North Shore Hospital, St. Leonards, NSW 2065 Australia; 2 Fidia Farmaceutici, S.p.A, Abano Terme, PD Italy

**Keywords:** Osteoarthritis, Synovial inflammation, Structural pathology, Pain, Mesenchymal stem cells, Hyaluronan

## Abstract

**Background:**

Synovitis is implicated in the severity and progression of pain and structural pathology of osteoarthritis (OA). Increases in inflammatory or immune cell subpopulations including macrophages and lymphocytes have been reported in OA synovium, but how the particular subpopulations influence symptomatic or structural OA disease progression is unclear. Two therapies, hyaluronan (HA) and mesenchymal stem cells (MSCs), have demonstrated efficacy in some clinical settings: HA acting as device to improve joint function and provide pain relief, while MSCs may have immunomodulatory and disease-modifying effects. We used these agents to investigate whether changes in pain sensitization or structural damage were linked to modulation of the synovial inflammatory response in post-traumatic OA.

**Methods:**

Skeletally mature C57BL6 male mice underwent medial-meniscal destabilisation (DMM) surgery followed by intra-articular injection of saline, a hyaluronan hexadecylamide derivative (Hymovis), bone marrow-derived stem cells (MSCs), or MSC + Hymovis. We quantified the progression of OA-related cartilage, subchondral bone and synovial histopathology, and associated pain sensitization (tactile allodynia). Synovial lymphocytes, monocyte/macrophages and their subpopulations were quantified by fluorescent-activated cell sorting (FACS), and the expression of key inflammatory mediators and catabolic enzyme genes quantified by real-time polymerase chain reaction (PCR).

**Results:**

MSC but not Hymovis significantly reduced late-stage (12-week post-DMM) cartilage proteoglycan loss and structural damage. Allodynia was initially reduced by both treatments but significantly better at 8 and 12 weeks by Hymovis. Chondroprotection by MSCs was not associated with specific changes in synovial inflammatory cell populations but rather regulation of post-injury synovial *Adamts4*, *Adamts5*, *Mmp3*, and *Mmp9* expression. Reduced acute post-injury allodynia with all treatments coincided with decreased synovial macrophage and T cell numbers, while longer-term effect on pain sensitization with Hymovis was associated with increased M2c macrophages.

**Conclusions:**

This therapeutic study in mice demonstrated a poor correlation between cartilage, bone or synovium (histo)pathology, and pain sensitization. Changes in the specific synovial inflammatory cell subpopulations may be associated with chronic OA pain sensitization, and a novel target for symptomatic treatment.

## Background

Osteoarthritis (OA) is the most prevalent of all joint diseases, with ageing, obesity, and injury identified as significant risk factors. Despite the strong age association, the majority (64%) of those affected by OA are of working age (15–64 years) accounting for 11% of the workforce [1]. OA is characterised clinically by pain and disability and pathologically by abnormalities in all joint tissues such as cartilage erosion and loss, thickened subchondral bone of reduced mineral density, excessive marginal new bone formation (osteophytes), and synovitis. Cartilage breakdown combined with its poor reparative capacity means that its loss in OA signifies “end-stage disease” and drives the need for joint replacement. However, numerous clinical studies have shown that incident and worsening pain in knee OA are much more strongly associated with subchondral bone marrow lesions and synovial inflammation/synovitis rather than with cartilage pathology [2–5]. Interestingly, synovitis, unlike bone marrow lesions, is also associated with neuropathy-like pain sensitization in knee OA patients [6].

In addition to its relationship with knee pain, synovitis is also strongly associated with more rapid progression of cartilage loss in OA joints and initiation of cartilage loss in joints without OA [7–10]. This suggests that synovial inflammation can occur upstream of cartilage erosive mechanisms and could therefore not only be a therapeutically accessible symptom- but also disease-modifying target. Data from genetically modified mice has confirmed that ablation/inactivation of specific inflammatory molecules/pathways reduces joint structural pathology, particularly cartilage degradation, in induced models of post-traumatic (pt)OA [11]. Similarly, there is evidence that depletion of macrophages [12, 13] and lymphocytes [14, 15] can ameliorate ptOA joint pathology in mice. While these studies implicate specific inflammatory/immune-response pathways in regulating ptOA synovitis and its downstream structural consequences, the relationship to pain and symptoms has not been well investigated.

The medial meniscal destabilisation (DMM) model of ptOA in mice induces pathology in all joint tissues along with pain/disability measures that mimic structural and symptomatic changes seen in patients [11, 16, 17]. Recently, the persistence of synovitis in DMM above that induced by sham-surgery has been described, along with a method to examine the inflammatory cell populations in mouse knee joint synovial tissues using fluorescence-activated cell sorting (FACS) [18]. Thus, DMM provides a validated model to evaluate the association between therapeutically modulating the synovial inflammatory response and changes in pain and/or pathology in ptOA. In this study, we tested two clinically used intra-articular therapies reported to modulate synovitis and ptOA pain but with different mechanisms of action: a hyaluronan (HA) derivative with extended joint residency (Hymovis®) and mesenchymal stem cells (MSCs). The viscoelastic properties of Hymovis modulate joint function and pain and reduce synovitis and synovial fibrosis in a large animal model of ptOA [19, 20]. MSCs are also reported to improve pain and function in ptOA, but this is largely thought to be as a result of secretion of anti-inflammatory and immunomodulatory factors, which may also modulate progression of structural pathology [21–24]. We determined the effect of these two agents alone or in combination on DMM-induced cartilage and bone pathology, synovitis, synovial inflammatory cell populations and gene expression, and pain sensitization. We evaluated whether modulation of any specific aspect of the ptOA-associated synovial inflammatory response was associated with symptomatic or structural disease modification.

## Material and methods

### OA induction and treatment

All procedures were approved by the Royal North Shore Hospital Animal Ethics Committee (protocol 1311-009A). All mice were from a C57BL6 colony at the Kolling Institute Kearns Facility established and replenished every 10 generations with mice from Jackson Labs. Animals (2–5 per 30 × 20 × 18 cm individually ventilated cage with filter lids, sterilised bedding, and environmental enrichment) received acidified water and a commercial complete pelleted food ad libitum and were maintained at 21–22 °C with a 12-h light/dark cycle. OA was surgically induced in 10–12-week-old males (*n* = 312) by unilateral (right) DMM as previously described [18, 25]. Male mice were used as they develop more robust and consistent OA and associated pain behaviours than female mice following DMM [26, 27]. Mice were randomly assigned to receive intra-articular injection (into the operated joint only) at 2 and 4 weeks post-DMM: 10 μl saline as the vehicle control (“Saline”), 10 μl Hymovis® (“Hymovis”), 2 × 10^4^ passage 6 heterologous mouse bone marrow-derived MSCs in 10 μl saline (“MSC”; cell dose based on previous studies using adipose-derived MSCs in mouse OA models [23, 24]), or 2 × 10^4^ MSCs at week 2 then Hymovis® at week 4 (“MSC + Hymovis”). Individual treatments were prepared in a laminar flow hood < 1 h prior to administration in disposable 0.3 ml insulin syringes with 31-gauge needles. The syringe contents were covered by tape and labelled A–D to blind the administrator and transported on ice to the animal facility. Mice were anaesthetised (2% isoflurane), placed in dorsal recumbency, the skin over the joint shaved and swabbed with ethanol, the knee extended and the needle inserted proximo-laterally through the distal medial aspect of the patella tendon (identifiable through the skin as a white band) until the tip sat in the trochlea groove beneath the patella, the contents injected, the needle removed, and the knee flexed and extended several times, before the mouse was removed from anaesthesia and recovered. Animals in each group were sacrificed at 4, 8 or 12 weeks post-surgery, after receiving intra-articular injections at weeks 2 and 4; the 4-week sacrifice group received a single dose at week 2, with the MSC+Hymovis group receiving both agents simultaneously.

### Mouse MSC isolation, expansion, and characterisation

Wild-type C57BL6 mice (10 × 6–8 week-old male) were euthanized, and bone marrow (femur and humerus) MSCs isolated and culture-expanded as described [28]. No plastic-adherent cells survived from three animals; those from the remaining seven mice were grown to fourth passage before being pooled, and frozen in 0.5 ml aliquots (DMEM/20% FBS/10% DMSO; 2 million cells/ml; − 80 °C). To prepare cells for injection, frozen aliquots were thawed, cultured to passage 6, and on the day of injection isolated by trypsin digestion, counted, and resuspended in sterile saline (2 × 10^6^ cells/ml). Equivalent passage 6 cells were analysed for cell surface markers (CD105, CD29, Sca1, CD45; R&D Systems Mouse MSC Kit FMC003) and tri-lineage differentiation [29].

### Tactile allodynia

Mice allocated to be sacrificed 12 weeks post-DMM had tactile allodynia measured by a single assessor (SZ) blinded to treatment, 1 week prior (time “0”), and at post-DMM week 2 (pre-injection), 4 (pre-injection), 8, and 12 (pre-euthanasia), using von Frey filaments and the up-down method to calculate 50% withdrawal threshold as previously described [30].

### Synovial tissue analysis

Knees were harvested immediately after sacrifice, and synovial tissues (anterior synovium, joint capsule, and fat pad) isolated by micro-dissection [18]. In six animals from each group, synovial tissues were snap-frozen in liquid nitrogen for RNA isolation and quantitative reverse transcription-polymerase chain reaction (qRT-PCR) analysis as detailed previously [18, 25, 31]. Briefly, a nanodrop spectrophotometer was used to quantify RNA and ensure equivalent sample quality, and a no-RT-qPCR performed to confirm the lack of contaminant genomic DNA. The same quantity (1 μg) of RNA from all samples underwent simultaneous RT, and expression of molecules implicated in OA pathophysiology (*Adamts4*, *Adamts5*, *Mmp2*, *Mmp3*, *Mmp9*, *Mmp13*, *Il1*, *Il6*, *Ccl3*) determined using mouse-specific primers (Table 1). Standard curves (fourfold dilutions of pooled mouse synovial cDNA) were included in each run. The threshold cycle for each gene was determined and converted to a relative fluorescence unit (RFU) by interpolation of the standard curve by the RotorGene qPCR software. This automated method enables detection of both PCR efficiency and high cycle inhibition, while providing similar values to delta-delta Ct methods [32]. Melt curves after each qPCR run verified a single gene-specific amplification product. As the “house-keeping” gene *Gapdh* was differentially regulated with both time and treatment (Fig. 4), genes of interest were corrected based on RT of equal total RNA [33] and expressed as “fold-change” by dividing each sample RFU by the mean 4-week Saline RFU.

**Table 1:** Selected genes and their mouse-specific primer sequences

Gene name	Accession number	Sequence	Melt (°C)	Product size
*Gapdh*	NM_001289706.1	F—TGCGACTTCAACAGCAACTC R—CTTGCTCAGTGTCCTTGCTG	55	200
*Adamts4*	NM_172845.3	F—TAACTTGAATGGGCAGGGGGGTTC R—AATGGCTTGAGTCAGGACCGAAGG	60	245
*Adamts5*	NM_011782.2	F—TCTCCAAAGGTTACGGATGGG R—TCTTCTTCAGGGCTAAGTAGGCAG	55	298
*Mmp2*	NM_008610	F—ATTTGGCGGACAGTGACACCAC R—ATCTACTTGCTGGACATCAGGGGG	59	231
*Mmp3*	NM_010809.1	F—GCTGAGGACTTTCCAGGTGTTG R—GGTCACTTTTTTGGCATTTGGGTC	53	120
*Mmp9*	NM_013599	F—TGGCTTTTGTGACAGGCACTTC R—CGGTGGTGTTCTCCAATGTAAGAG	55	223
*Mmp13*	NM_008607	F—GATGACCTGTCTGAGGAAG R—ATCAGACCAGACCTTGAAG	55	357
*IL1b*	NM_008361.4	F—ACCTGTTCTTTGAAGTTGACGGAC R – TCTTGTTGATGTGCTGCTGCGAG	55	117
*IL6*	NM_031168.2	F—CTTCCATCCAGTTGCCTTCTTG R – TGTTGGGAGTGGTATCCTCTGTG	55	104
*Ccl3*	NM_011337	F—GAAGGATACAAGCAGCAGCGAG R—GAGAAGAACAGCAAGGGCAGTG	55	115

Synovial tissues from the remaining 20 animals in each group were used for FACS analysis: tissues from 4 mice pooled to provide sufficient cells for analysis, enabling evaluation of 5 “biological replicates” from 20 mice [18]. Compensation settings for spectral overlap on the multi-colour flow cytometer were set using the AbC Total Antibody Compensation Kit (Thermo Fischer), and isotype control antibodies were included in every FACS run. Viable (7-aminoactinomycin-D negative) cells were gated on forward/side scatter and quantified using FlowJo software (Tree Star) as CD3^+^CD4^+^CD8^−^ T helper cells, CD3^+^CD4^−^CD8^+^ cytotoxic T cells, CD11b^+^F4/80^−^Ly6c^+^ resident monocytes, CD11b^+^F4/80^+^Ly6c^high^ inflammatory monocytes, and CD11b^+^F4/80^+^Ly6c^low^ activated/inflammatory macrophages. The macrophages were further characterised as M1 (F4/80^+^CD11c^+^) and M2 (F4/80^+^CD206^+^) subtypes, with the latter further identified as “M2a wound healing” (CD206^+^CD150^+^) and “M2c anti-fibrotic” (CD206^+^CD301^+^).

### Histopathology

The knee joint tissues remaining after removal of anterior synovium (described above) were fixed, decalcified, paraffin-embedded, and serial sagittal sections across the medial femoro-tibial joint cut and stained with toluidine blue-fast green as previously described [18, 25]. Maximum and cumulative (total in all sections) scores of femoral and tibial cartilage proteoglycan loss and structural damage, plus tibial subchondral bone sclerosis, anterior tibial osteophyte size and maturity, and posterior femoral synovitis, were determined by two independent observers (CS, VR) blinded to time and treatment, and a mean value for each histopathological feature in each joint generated [18, 25].

#### Statistical analyses

The number of animals required in each group was determined prior to study initiation, based on prior data using the same mouse model and methods [18, 25, 34]. The number of animals required to achieve > 85% power differs with the outcome measure: 12 animals to detect a clinically meaningful difference of > 30% in allodynia; *n* = 5 FACS replicates allowing detection of 60% change in cell numbers, 40% change in the percentage of macrophages, and twofold difference in the percentage of T cells; *n* = 18 is required to detect changes of 25–60% in different histopathology scores; and *n* = 6 enables RT-PCR differences of 1.1–5.8-fold (depending on the gene analysed) to be detected. As some data was non-continuous (histopathology) and not normally distributed (allodynia, FACS, histopathology), differences between treatments within a time point and for a given treatment over time were compared using a rank-sum test for nonparametric data (Stata 12). In this exploratory study, no multiple comparisons correction was applied, and *P* < 0.05 was considered statistically significant.

## Results

All animals successfully completed the study, with no adverse events observed from surgery or intra-articular injection, and data from all animals was included in the analysis, i.e. no removal of outliers.

### Isolation and characterisation of mouse MSCs

**Fig. 1:**
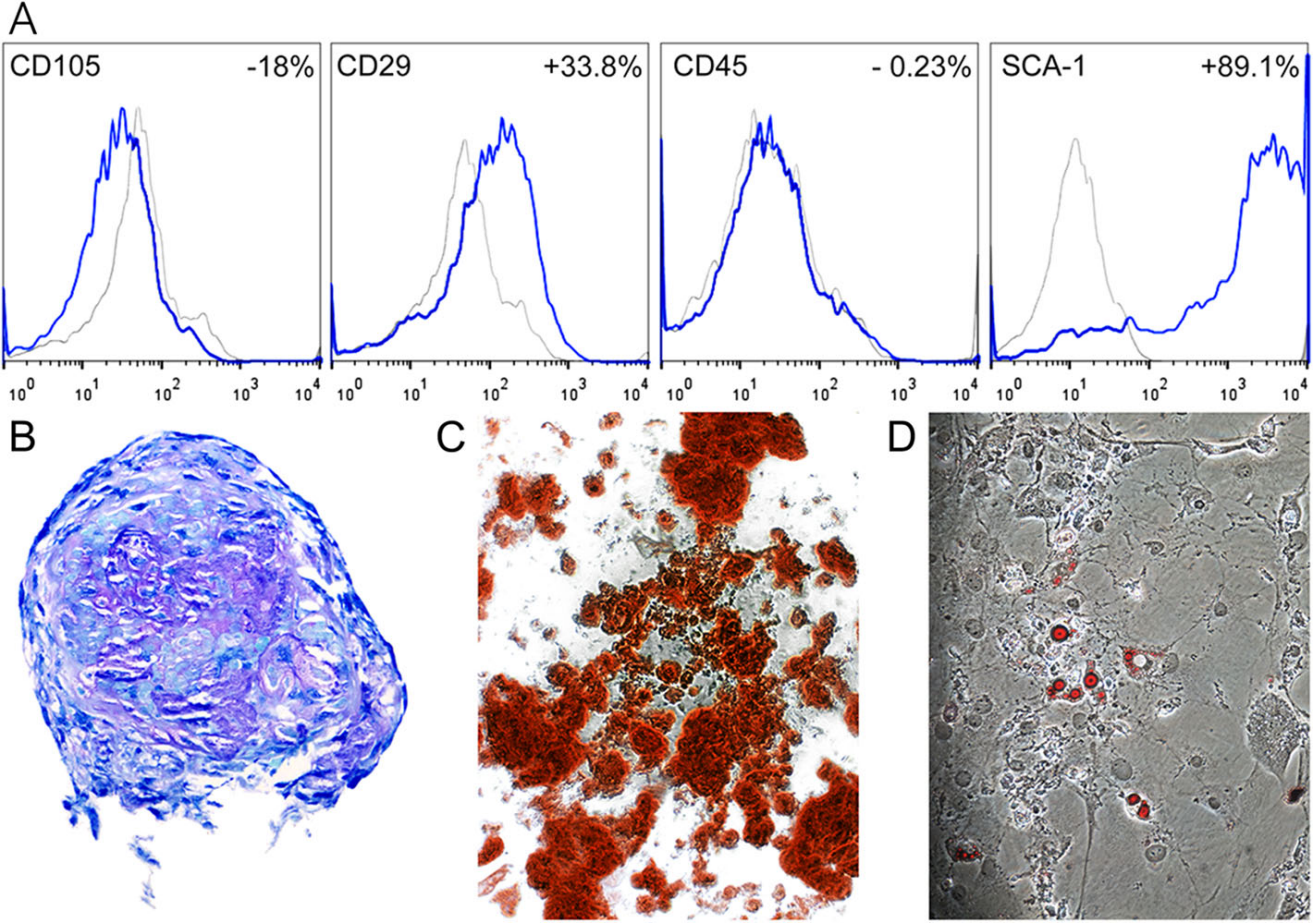
**a** FACS analyses of passage 6 mouse bone marrow-derived stromal cells for stem cell surface markers (CD105, CD29, Sca-1, and CD45). Grey indicates sotype controls, and blue indicates cells stained for specific cell surface markers. The percentage of positive staining cells compared to respective isotype control is shown for each cell surface marker. Representative images demonstrating **b** chondrogenic (metachromatic toluidine blue staining of glycosaminoglycan accumulation), **c** osteogenic (alizarin red staining of calcium deposition), and **d** adipogenic (oil red O staining of intracellular lipid) differentiation of pooled P6 bone marrow-derived stromal cells

FACS analyses of passage 6 plastic-adherent cells demonstrated that they were CD45 negative (Fig. 1a) confirming the lack of haematopoietic lineage. We were unable to demonstrate CD105 reactivity but had > 33% CD29 and > 89% Sca1-positive cells, and demonstrated chondrogenic, osteogenic, and adipogenic differentiation (Fig. 1b–d).

### Tactile allodynia

**Fig. 2:**
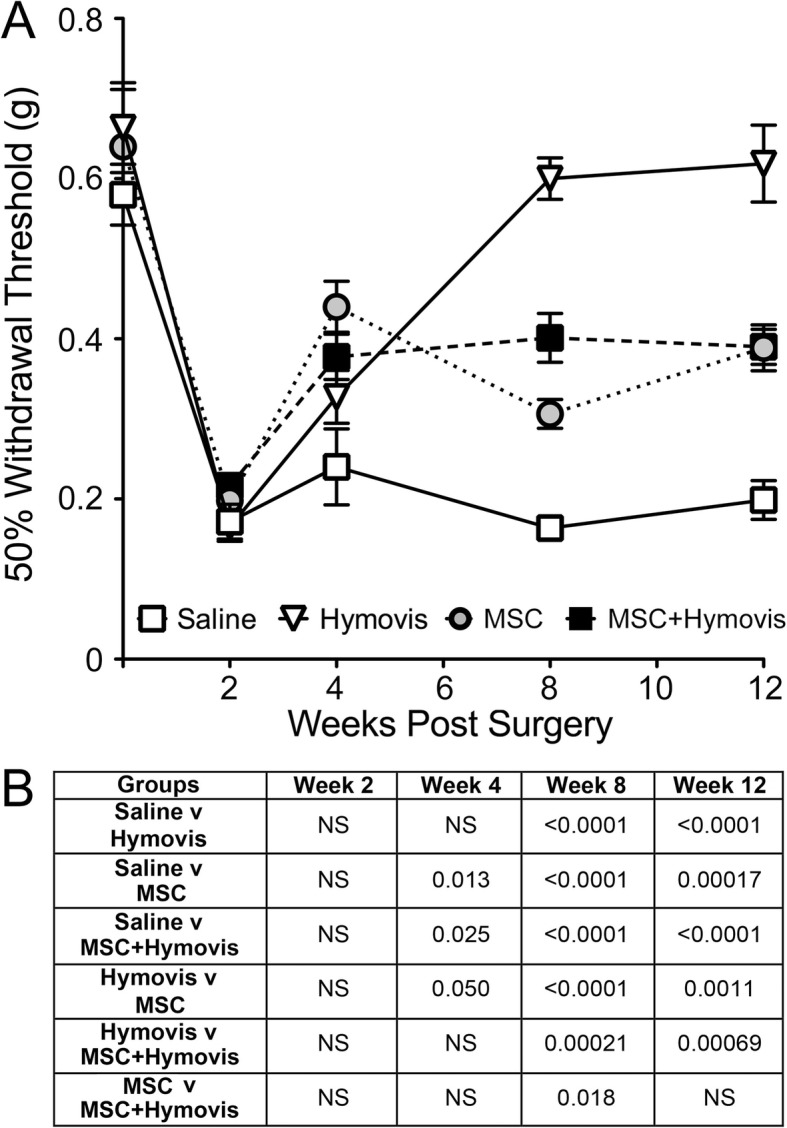
**a** Tactile allodynia (50% paw withdrawal threshold; mean ± SEM) in mice measured serially over time using von Frey fibres. A single observer blinded to treatments performed the measurements: Saline (white squares), Hymovis (white triangles), MSC (grey circles), and combined MSC + Hymovis (black squares). **b** Table of *P* values for comparisons between treatments at specific time points (NS = not significant)

There was no difference in withdrawal threshold between any group pre-surgery (Fig. 2). Mechanical allodynia was evident post-DMM in all groups (2 weeks), with this same level of reduced withdrawal threshold maintained throughout the study for the Saline group (*P* < 0.0001 for baseline versus all subsequent times). Withdrawal threshold was not significantly improved after the first dose of Hymovis compared with Saline but was dramatically ameliorated following the second injection where it returned to baseline levels at week 8–12. Allodynia was significantly reduced at week 4 after a single MSC injection ± Hymovis. A second dose of MSCs at week 4 was followed by worsening of allodynia at week 8 (*P* < 0.01) although it still remained improved compared with Saline, and then returned to week 4 levels by week 12. When MSC injection at week 2 was followed by Hymovis at week 4, there was no change in allodynia with withdrawal threshold improved compared with week 2 (*P* < 0.0005 between 2 and 4, 8, and 12 weeks), and significantly better than saline. OA-associated allodynia at weeks 8 and 12 following treatment with two doses of MSCs or MSCs followed by Hymovis never improved to the level seen with two injections of Hymovis.

### Mouse OA histopathology

**Fig. 3:**
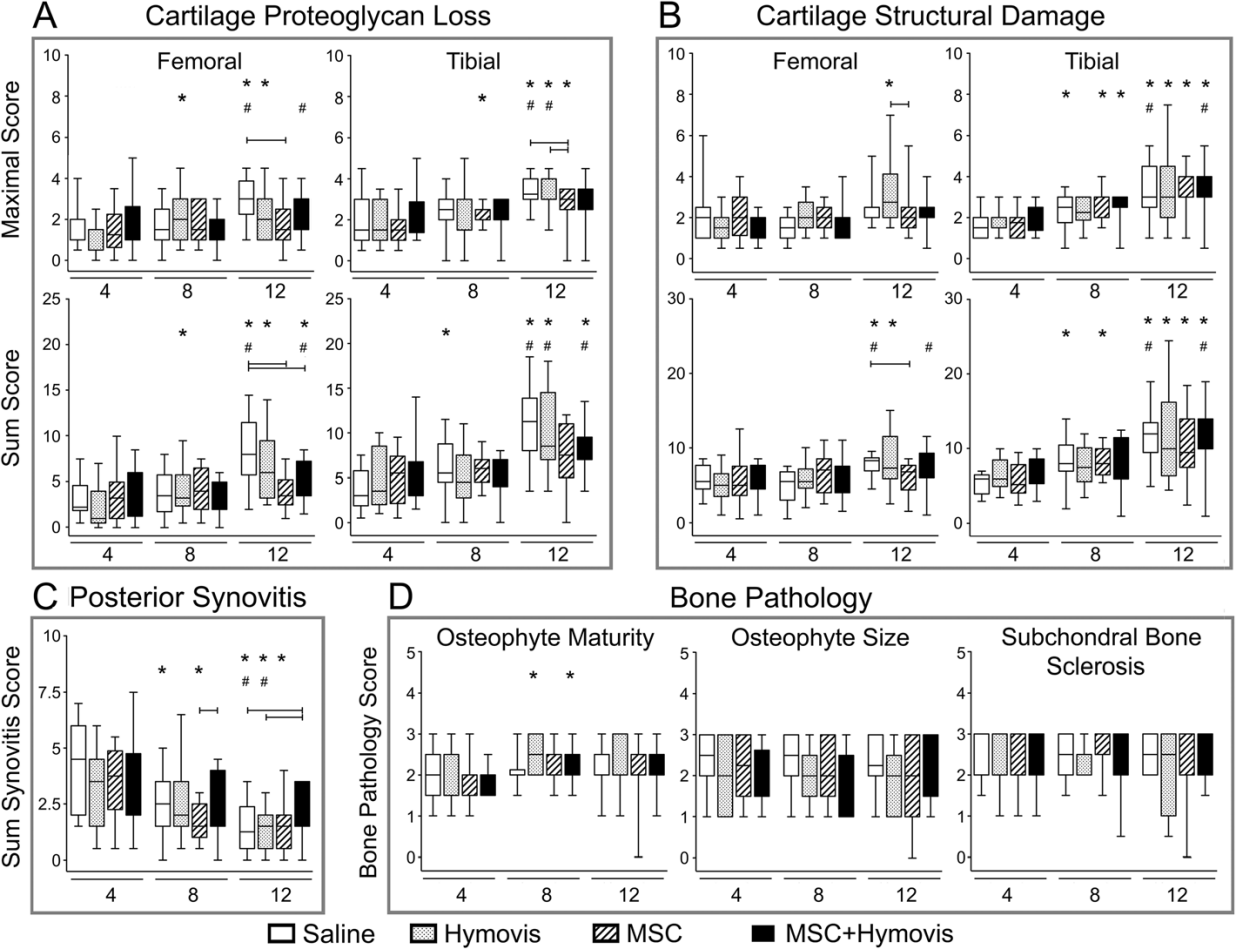
Histology scoring of OA pathology at different 4, 8 and 12 weeks post-DMM from mouse OA joints (box plot showing 25–75% percentile [box], median [line in the box], and data range [whiskers]). Maximal scores (single highest pathology score per joint) and summed scores (sum of scores from all the slides per joint) are presented. **a** Cartilage proteoglycan loss. **b** Cartilage structural damage. **c** Posterior synovitis. **d** Bone pathology. Brackets indicate *P* < 0.05 between treatments at the same time point. *P < 0.05 compared with the same treatment group 4-week scores. ^#^*P* < 0.05 compared with the same treatment group 8-week scores

Maximal and summed cartilage proteoglycan loss in the femur and tibia increased from 4 to 12 weeks (Fig. 3a). Although no difference was observed at 4 or 8 weeks, median proteoglycan loss in the MSC (*P* < 0.05 femoral and tibial maximal score, femoral sum score) and MSC + Hymovis (*P* < 0.05 femoral sum score) groups was reduced compared to Saline at 12 weeks. Cartilage structural damage particularly in the tibia increased with time with all groups (Fig. 3b), with the femoral summed score at 12 weeks reduced in the MSC group compared with Saline (*P* < 0.05). Synovitis at 4 weeks showed no difference between treatments, and scores reduced from week 4 through 12 in all except MSC + Hymovis, which by week 12 was significantly increased compared with Saline and Hymovis. No differences in subchondral bone pathology or osteophyte development were observed with time, and there was no difference with or between any treatment (Fig. 3d).

### Synovial gene expression

**Fig. 4:**
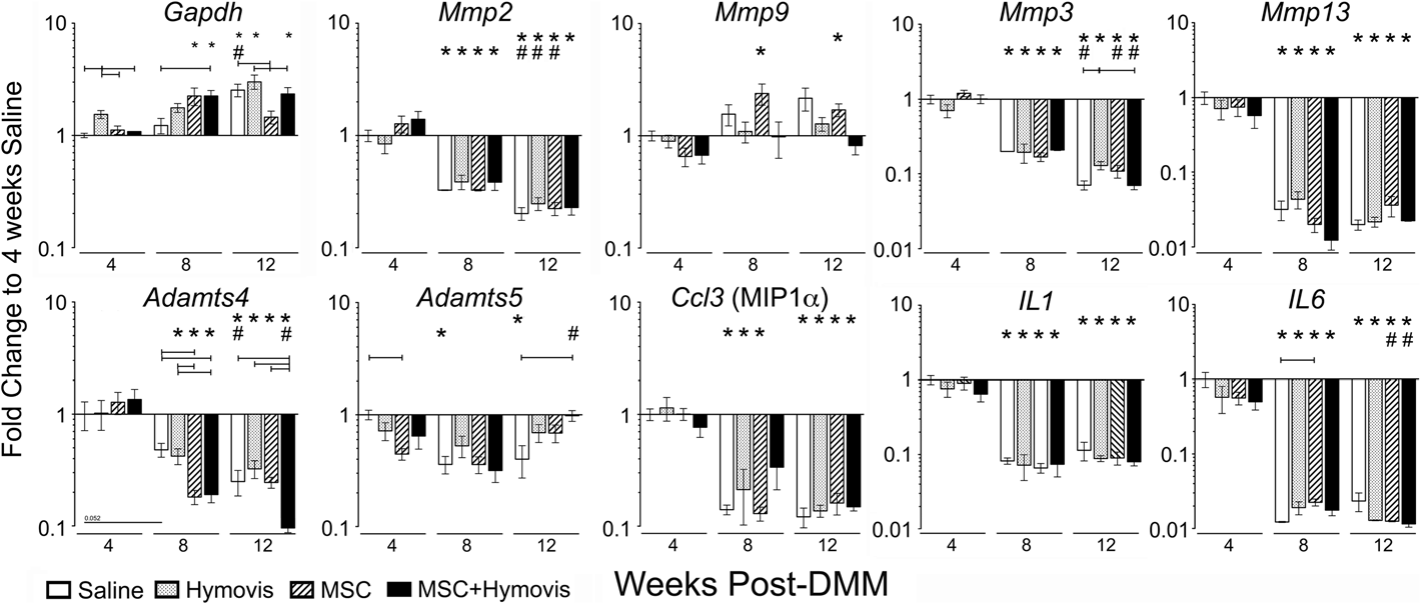
Gene expression in mouse synovial tissues at different times post-DMM presented as fold change compared with 4-week Saline sample (mean ± SEM; *n* = 6 per treatment and time point). Brackets indicate *P* < 0.05 between treatments at the same time point. *P < 0.05 compared with the same treatment group 4-week expression. ^#^*P* < 0.05 compared with the same treatment group 8-week expression

Expression of *Mmp2*, *Mmp3*, and *Mmp13* reduced over time, with the only differences between treatments being higher *Mmp3* at week 12 in MSC (Fig. 4). In contrast, *Mmp9* expression increased in Saline and MSC (but not Hymovis or MSC + Hymovis) at weeks 8 and 12 compared with week 4. *Adamts4* decreased significantly with time, and while unaffected by treatment at week 4, was reduced in MSC and MSC + Hymovis compared to Saline and Hymovis at week 8, and by week 12 was further inhibited in MSC + Hymovis (*P* < 0.05 to all other treatments). *Adamts5* expression was significantly decreased in MSCs versus Saline at week 4. The significant decrease in *Adamts5* in Saline by week 8 meant there was no difference between treatment groups at this time. By week 12, *Adamts5* expression in all groups other than Saline generally returned to week 4 levels, particularly MSC + Hymovis which was significantly greater than Saline. All three inflammatory cytokines showed a significant time-dependent decrease in expression after week 4. There was no difference between treatments in the temporal decrease or between treatments at any time point for *Il1* or *Ccl3* (MIP1α—macrophage inflammatory protein alpha)*. Il6* expression, however, was significantly greater in MSC compared with saline at week 8 but was not different between treatments at any other time.

### Synovial inflammatory cell flow cytometry

**Fig. 5:**
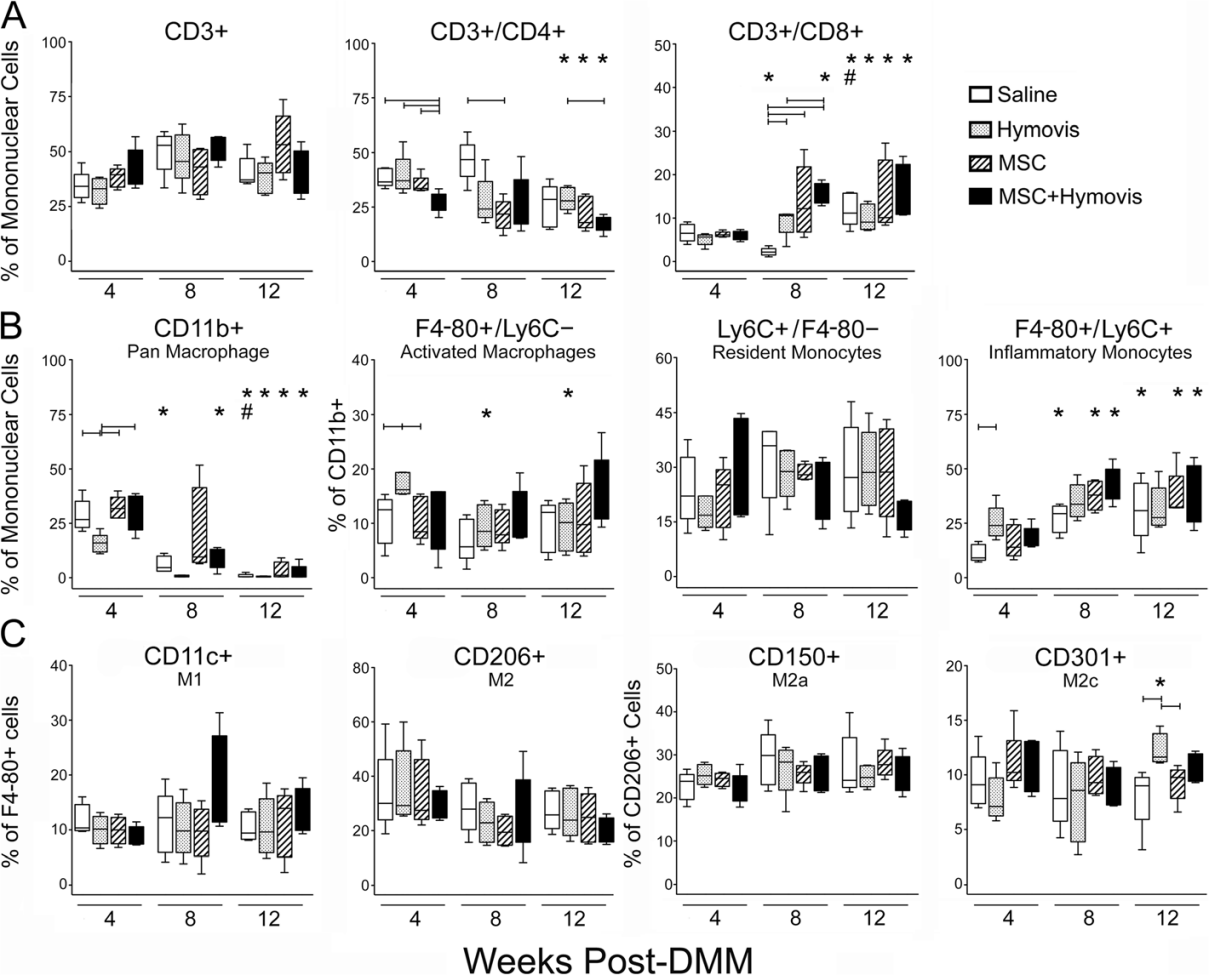
Fluorescence-activated cell sorting (FACS) analysis of synovial tissue inflammatory cell populations at 4, 8, and 12 weeks post-DMM. **a** Lymphocytes. **b**, **c** monocytes and macrophages. Data is presented as either a percentage of the total mononuclear cells (**a**, **b**) or a specific subpopulation of cells (**b**, **c**), as box plots showing 25–75% percentile (box), median (line in the box), and data range (whiskers). Brackets indicate *P* < 0.05 between treatments at the same time point. *P < 0.05 compared with the same treatment group at 4 weeks. ^#^*P* < 0.05 compared with the same treatment group at 8 weeks. Note the different *Y*-axes scale on each graph for better data illustration

The total number of lymphocytes (CD3^+^) and monocyte/macrophage lineage cells (CD11b^+^) at 4 weeks post-DMM was significantly and equally reduced by all treatments compared with saline (Additional file 1: Table S1). Total CD3^+^ lymphocyte number in saline-treated joints decreased after 4 weeks such that there was no difference compared with other treatments at 8 or 12 weeks. Monocyte/macrophage lineage (CD11b^+^) cell number decreased significantly in all groups after 4 weeks, but the reduction was greater in Saline such that numbers did not differ compared with other treatments at 8 or 12 weeks. This cell number data reflects the burden of a particular cell type and demonstrated a significant acute beneficial effect of MSCs, Hymovis, and MSC + Hymovis. However, differences in total viable mononuclear cell numbers between groups may mask more subtle effects of individual treatments on subpopulations and activation of lymphocytes and monocytes/macrophages. This is better revealed by evaluating a particular cell type as a percentage of either the total mononuclear cells or a given inflammatory cell population (Fig. 5). While changes in the percentage of any cell type may be artificially magnified with low absolute numbers, in almost all cases, counts were > 100 (and usually much more) for any specific cell type gate even at 12 weeks (Additional file 1: Table S1).

While CD3^+^ lymphocytes represented a similar percentage of the synovial mononuclear cell population at all times in all treatments, the percentage of these that were CD4^+^ versus CD8^+^ differed with both time and treatment (Fig. 5a). The percentage of CD4^+^ T cells decreased with time earlier and by 12 weeks to a greater extent in all treated groups (*P* < 0.05 compared with week 4) compared with Saline. In contrast, the percentage of CD8^+^ T cells increased in all mice with time, again more rapidly in treated compared with Saline joints (Saline < all treatments at week 8 (*P* < 0.05) but not by week 12). Monocyte lineage (Cd11b^+^) cells as a percentage of synovial mononuclear cells were reduced by Hymovis at week 4 (*P* < 0.05 versus all other groups), and a significantly greater percentage of these CD11b^+^ cells were activated (F4/80^+^) in Hymovis-treated joints compared with Saline (*P* < 0.05; Fig. 5b). There was no difference between groups in macrophage polarisation (M1/M2; M2a/M2c) at 4 weeks (Fig. 5c). With time post-DMM, the percentage of mononuclear cells that were CD11B^+^ decreased in all groups (*P* < 0.05 week 12 versus week 4), although this was delayed in mice receiving two doses of MSCs which showed no significant decrease at week 8 (Fig. 5b). There was little change with time post-DMM in the percentage of CD11B^+^ cells identified as resident monocytes (Ly6C^+^F4/80^−^) or activated macrophages (F4/80^+^Ly6C^−^), other than with Hymovis treatment where they were elevated at week 4 but returned to levels seen in all other joints thereafter. The percentage of CD11B^+^ cells identified as inflammatory monocytes (F4/80^+^Ly6C^+^) increased with time in all joints other than Hymovis-treated (which was already elevated at 4 weeks) such that there was no difference between groups at week 8 or 12 post-DMM. There was no significant change with time or difference between groups at any time in the percentage of F4/80^+^ macrophages identified as M1 (CD11C^+^) or M2 (CD206^+^) subtypes (Fig. 5c). However, the percentage of anti-fibrotic M2c macrophages (F4/80^+^CD206^+^CD301^+^) increased at week 12 in Hymovis-treated joints to be significantly greater than Saline and MSC-treated joints at this time (Fig. 5c).

## Discussion

This study in a mouse model of ptOA has confirmed our previous findings in sheep that intra-articular Hymovis can significantly reduce measures of OA pain [19]. In the current study, we found that two doses of Hymovis (2 weeks apart) was significantly better at reducing chronic mechanical allodynia than two intra-articular MSC injections, or MSCs followed by Hymovis, although these two treatment protocols were still superior to placebo. Interestingly, for none of the treatments was the reduction in allodynia temporally associated with improvement in histopathological measures of OA structural pathology, including cartilage erosion, subchondral bone remodelling, or osteophytes. This reflects the generally poor relationship between radiographic OA structural pathology and pain in humans [3]. In fact we observed a clear disconnect in the association between structural-pathology and pain, with the superior effect of Hymovis on allodynia occurring without significant histopathological disease modification in any joint tissue, while MSCs (alone or followed by Hymovis) significantly reduced late cartilage degradation but had less effect on pain sensitization. Symptom modification without concomitant structural protection is also consistent with our previous observations with Hymovis in a sheep OA model [19] and a recent study of different hyaluronan preparations in mouse DMM [35]. The superior effect on allodynia with Hymovis in the current study may be associated with direct effects of hyaluronan elastoviscous properties on reducing pain-elicited nerve activity [36].

There was a significant beneficial effect of MSCs, particularly with 2 consecutive doses, on later-stage (12 weeks) progression of cartilage proteoglycan loss and erosion. While reduced proteoglycan loss was seen in both the femur and tibia, this was accompanied by reduced structural damage only in the femur. This may be indicative of a better effect of MSCs on less severe chondropathy, as is typically observed in the femur compared with tibia in the DMM model [34, 37]. A similar explanation was proposed for the site-specific chondroprotection in OA patients treated with FGF18, where beneficial effects were seen in the more mildly affected lateral but not the more severely affected medial femorotibial compartment [38]. Alternatively, the OA molecular pathophysiology of cartilage degradation may be different in femoral compared with tibial cartilage, as is suggested by topographically distinct patterns of gene expression [39, 40], and this could lead to regionally distinct therapeutic responses.

There are mixed reports on the effect of intra-articular MSCs on OA structural pathology, potentially related to differences in cell source (e.g. autologous versus heterologous, marrow- versus adipose-derived, directly isolated stromal cells versus culture-expanded), cell number, carrier, timing of administration, outcome measures evaluated, and animal model used, amongst others [21, 41, 42]. Our data showing chondroprotective effects of heterologous marrow-derived culture-expanded MSCs contrasts with a recent study using adipose-derived MSCs in the same DMM model [23]. Whether this relates to bona fide differences in therapeutic efficacy between MSCs derived from different tissues is unclear. MSCs are defined by the characteristics of self-renewal and multi-potent differentiation; however, cells from different strains of mice [43], different tissues [44], and tissue sites [45] within mice, and even subpopulations within a single tissue [46], show variability in their differentiation potential and associated cell-surface markers. The multi-potent MSCs used in the current study had poorer adipogenic compared with their osteo- and chondro-genic ability, and despite being ~ 90% positive for the MSC marker Sca-1 (similar to previous OA-therapeutic studies using MSCs from bone marrow [43] or adipose tissue [23, 24]), they had only modest CD29 expression and were negative for CD105. This is consistent with neither the relative differentiation potential nor specific cell surface markers being predictive of MSC therapeutic efficacy in post-traumatic OA [43]. Along with cell differences that may account for divergent results, it is noteworthy that the previous study only evaluated animals 8 weeks post-DMM [23], at which time we also found no structural protection in MSC-treated joints. The reason for the potentially delayed protective effect of MSCs we observed is not clear but could be associated with changes in the molecular pathophysiology of cartilage degradation with time post-DMM [47, 48].

While incident and worsening pain in patients with knee OA is consistently associated with imaging measures of synovitis and effusion [2–6], the correlation between clinical symptoms and synovial biopsy histology in established disease is poor [49]. This may be driven by the focal nature of OA synovitis whereby lesions are missed in small biopsies. In the current mouse model, the complete posterior synovium was available for scoring, and while less affected acutely after surgery than anterior tissues, this region does show persistently increased inflammation (week 1–16 post-DMM) compared with both naive joints and sham surgery [18]. Thus, synovitis evident histologically after DMM is temporally associated with the onset and persistence of allodynia, akin to synovitis-imaging-pain association in patients. Consistent with patient data where significant improvement in symptoms with treatments such as intra-articular corticosteroids is not correlated with reduced imaging measures of synovitis [5], the beneficial therapeutic effect of MSCs and Hymovis on allodynia was not associated with improvement in histopathological synovitis in our mice. Histopathology, like imaging, may not be suitable for detecting subtle differences in synovial inflammation, motivating our more in-depth analysis using PCR and FACS.

There were a limited number of treatment-specific synovial gene expression changes that might be associated with the variable therapeutic effects on allodynia and chondroprotection. There was evidence that Hymovis but not MSCs ameliorated later-stage OA-associated synovial *Mmp9* upregulation. MMP9 is one of the key synovitis hub genes recently identified in human OA [50] and known to play a role in nociceptor sensitization [51]. In contrast, MSCs particularly when injected twice slowed the natural post-injury resolution of another of the key OA synovitis hub genes, *Il6*, which may in part explain their reduced effect on allodynia. It was notable that MSCs (either injected twice or once followed by Hymovis) induced a significantly greater reduction in synovial *Adamts4* expression at week 8 compared with Saline or Hymovis, and the MSC + Hymovis combination therapy further reduced *Adamts4* at week 12. Synovial fluid ADAMTS4 activity is correlated with synovial inflammation in patients undergoing arthroscopy [52]; thus, the decrease in *Adamts4* mRNA we observed may be indicative of a specific modulation of the inflammatory response by MSCs. The reduction in synovial *Adamts4* by MSCs was associated with a significant reduction in cartilage proteoglycan loss and cartilage erosion (particularly in the femur) at later time points. ADAMTS4 KO mice have not demonstrated a reduction in cartilage structural damage post-DMM; however, these previous studies did not separately evaluate femur and tibia and only examined mice up to 8 weeks post-DMM [53, 54] at a time point at which we also observed a lack of chondroprotection. While a single dose of MSCs did cause a more rapid decline in *Adamts5* expression, differences compared with Saline were not maintained. It is interesting to speculate that while ADAMTS5 is clearly the predominant aggrecanase responsible for acute aggrecanolysis in mouse cartilage in vitro and in different in vivo arthritis models [55, 56], perhaps synovial ADAMTS4 plays a role in later stages of disease.

While we found significant therapeutic modulation of synovial inflammatory/immune cells, a relationship with differential effects on pain was less clear. Hymovis, MSCs, and the combination induced equivalent reduction in lymphocyte numbers compared with Saline at 4 weeks in association with a similar reduction in allodynia in all treated mice at the same time point. This may suggest a role for synovial T cells in the mechanical allodynia that developed acutely joint injury and is consistent with the known role of T cells in regulating pain behaviours including mechanical allodynia in mice [57]. While CD4^+^ T cells have been strongly implicated in chronic neuropathic pain pathophysiology, the role of CD8^+^ T cells is less clear with both pro- and anti-algesic effects reported [58, 59]. The relative proportion of CD4^+^ and CD8^+^ T cells in all treatment groups at 4 weeks was largely not different compared with Saline, suggesting similar “broad” acute anti-inflammatory effects. At later times, however, MSCs had a more pronounced effect in reducing the percentage CD4^+^ T cells despite effects on allodynia from 8 to 12 weeks being significantly better in Hymovis. This is not consistent with findings in patients with late-stage OA where the percentage of CD4^+^ T cells in synovial tissues is associated with their pain [60]. In our study, all three treatments similarly increased CD8^+^ T cells in progressive OA (at 8 weeks) compared with Saline, which was again inconsistent with the divergent effects on allodynia at this time, and at 12 weeks, CD8^+^ T cells were not different in the three treatments compared with Saline despite clear differences in allodynia. Future work defining how subsets of CD4^+^ and CD8^+^ cells (e.g. Th1, Th2, Th17, Treg) are regulated in this OA model with time and treatment and how they relate to pain may be useful. Furthermore, there is evidence that T cells may play a greater role in chronic pain hypersensitivity in females [57], and while we used males for their more robust OA pathology and pain response following DMM, future studies should examine female mice.

All treatments also reduced the number of synovial monocyte/macrophage cells at 4 weeks in association with reduced allodynia. Hymovis had a greater effect in decreasing the percentage of CD11b^+^ cells but also increased their activation (F4/80^+^) at week 4, which was associated with a somewhat reduced effect on allodynia at this time compared with MSCs. As with lymphocytes, there was little or no treatment effect on monocytes/macrophages or their activation/polarisation at week 8 or 12, despite there being clear differentiation in allodynia at these times. Evaluation of the secretory profile of the activated monocytes/macrophages with time and treatment is needed to further understand their role in OA symptom development. Notably, in later-stage disease (12 weeks), Hymovis-treated joints had a significantly higher percentage of M2c macrophages. Within the M2 population, M2a are considered “pro-fibrotic” and M2c “anti-fibrotic” wound healing subtypes [61, 62]. The increased M2c subtype with Hymovis treatment is consistent with reduced joint capsule fibrosis seen in our previous sheep OA studies [20] which may contribute to its better long-term effect on pain sensitization [63].

The findings in the present study must be interpreted with consideration of a number of limitations. While a strength of the study is its evaluation of numerous outcomes and time points, we did not correct for multiple comparisons in our statistical analysis and future hypothesis-testing studies should be done. Nevertheless, our study was suitably powered based on a priori analysis using existing data. As already noted, we only used male mice and differences in response may be seen in females. Our animals while skeletally mature were young, equivalent to mid-late adolescent humans, and while this represents the greatest at-risk population, it is recognised that the molecular and cellular/inflammatory pathophysiology of subsequent ptOA may be different in older individuals [64, 65]. We have already noted the potential for different therapeutic response depending on MSC source, but the dose and timing of treatment may also be critical. Our FACS analysis methodology using bulk synovial tissue provides quantitative data on inflammatory cell types not possible with focal qualitative immunohistology. However, the need to pool tissues from four individuals to get sufficient cells means powerful direct correlation analysis between particular cell types and pathology or pain in an individual was not possible.

## Conclusions

This therapeutic study in a mouse model of ptOA has demonstrated a poor association between (histo)pathology in cartilage, bone or synovium, and pain sensitization. Pain sensitization in later-stage ptOA was significantly better controlled by early treatment with hyaluronan compared with MSCs, while only the latter provided long-term chondroprotection. A combination of the two treatments at least in the order tested in the current study (MSCs at week 2 followed by Hymovis at week 4) diminished the long-term structure and symptom-modifying benefits of two MSC or two Hymovis injections respectively, suggesting there may be benefit from the repeated individual treatments. Structural ptOA disease modification (with MSCs) was not associated with specific changes in synovial tissue inflammatory cell populations, but rather with the post-injury synovial metalloproteinase (*Adamts4*, *Adamts5*, *Mmp3*, *Mmp9*) expression. In contrast, reduced early (4 weeks) post-injury allodynia in response to intra-articular treatment with MSCs or Hymovis coincided with a decrease in the number of macrophages and T cells infiltrating the synovia tissues, with a higher percentage of activated macrophages possibly associated with a lesser analgesic effect at this time. Superior longer-term effects on pain sensitization in ptOA seen with the hyaluronan therapy may be associated with an increase in the percentage of anti-fibrotic M2c macrophages in the synovium and joint capsule. Future studies targeting specific inflammatory cell populations at different times after DMM (e.g. with intra-articular antibodies) may provide novel approaches to ptOA treatment.

## Supplementary information

**Additional file 1: Table S1.** Fluorescence activated cell sorting (FACS) analysis of the number (Mean ± SEM) of different synovial tissue inflammatory cell subtypes at 4, 8 and 12 weeks post-DMM (*n* = 5/treatment/time). Numbers highlighted in bold are significantly different (*P*<0.05) than for the same cell subtype in saline treated animals at that same time. Numbers marked with an * in weeks 8 and 12 are significantly different (P<0.05) than for the same cell subtype at 4 weeks in that treatment group. Numbers in italic text in week 12 are significantly different (P<0.05) than for the same cell subtype at 8 weeks in that treatment group.

## Data Availability

Data generated from this work and presented in this manuscript is publically available (DOI: 10.25833/e1yj-2q62) or is available upon request from the corresponding author.

## Abbreviations

OA: Osteoarthritis
ptOA: Post-traumatic OA
HA: Hyaluronan
MSC: Mesenchymal stem cell
DMM: Destabilization of the medial meniscus
FACS: Fluorescent-activated cell sorting
PCR: Polymerase chain reaction
DMEM: Dulbecco’s modified Eagles medium
FCS: Foetal calf serum
DMSO: Dimethyl sulfoxide

## References

[1] Access-Economics (2007). Painful realities: the economic impact of arthritis in Australia in 2007. Access Econmics Report.

[2] Mathiessen A, Conaghan PG (2017). Synovitis in osteoarthritis: current understanding with therapeutic implications. Arthritis Res Ther.

[3] Neogi T: Structural correlates of pain in osteoarthritis. Clin Exp Rheumatol 2017, 35 Suppl 107(5):75–78. 28967355

[4] O'Neill TW, Felson DT (2018). Mechanisms of osteoarthritis (OA) pain. Curr Osteoporos Rep.

[5] Wang X, Hunter DJ, Jin X, Ding C (2018). The importance of synovial inflammation in osteoarthritis: current evidence from imaging assessments and clinical trials. Osteoarthr Cartil.

[6] Neogi T, Guermazi A, Roemer F, Nevitt MC, Scholz J, Arendt-Nielsen L, Woolf C, Niu J, Bradley LA, Quinn E (2016). Association of joint inflammation with pain sensitization in knee osteoarthritis: the multicenter osteoarthritis study. Arthr Rheumatol.

[7] Roemer FW, Guermazi A, Felson DT, Niu J, Nevitt MC, Crema MD, Lynch JA, Lewis CE, Torner J, Zhang Y (2011). Presence of MRI-detected joint effusion and synovitis increases the risk of cartilage loss in knees without osteoarthritis at 30-month follow-up: the MOST study. Ann Rheum Dis.

[8] Roemer FW, Zhang Y, Niu J, Lynch JA, Crema MD, Marra MD, Nevitt MC, Felson DT, Hughes LB, El-Khoury GY (2009). Tibiofemoral joint osteoarthritis: risk factors for MR-depicted fast cartilage loss over a 30-month period in the multicenter osteoarthritis study. Radiology.

[9] Hill CL, Hunter DJ, Niu J, Clancy M, Guermazi A, Genant H, Gale D, Grainger A, Conaghan P, Felson DT (2007). Synovitis detected on magnetic resonance imaging and its relation to pain and cartilage loss in knee osteoarthritis. Ann Rheum Dis.

[10] de Lange-Brokaar BJ, Ioan-Facsinay A, Yusuf E, Kroon HM, Zuurmond AM, Stojanovic-Susulic V, Nelissen RG, Bloem JL, Kloppenburg M (2016). Evolution of synovitis in osteoarthritic knees and its association with clinical features. Osteoarthr Cartil.

[11] Little CB, Hunter DJ (2013). Post-traumatic osteoarthritis: from mouse models to clinical trials. Nat Rev Rheumatol.

[12] Blom AB, van Lent PL, Holthuysen AE, van der Kraan PM, Roth J, van Rooijen N, van den Berg WB (2004). Synovial lining macrophages mediate osteophyte formation during experimental osteoarthritis. Osteoarthr Cartil.

[13] Blom AB, van Lent PL, Libregts S, Holthuysen AE, van der Kraan PM, van Rooijen N, van den Berg WB (2007). Crucial role of macrophages in matrix metalloproteinase-mediated cartilage destruction during experimental osteoarthritis: involvement of matrix metalloproteinase 3. Arthritis Rheum.

[14] Shen PC, Wu CL, Jou IM, Lee CH, Juan HY, Lee PJ, Chen SH, Hsieh JL (2011). T helper cells promote disease progression of osteoarthritis by inducing macrophage inflammatory protein-1gamma. Osteoarthr Cartil.

[15] Hsieh JL, Shiau AL, Lee CH, Yang SJ, Lee BO, Jou IM, Wu CL, Chen SH, Shen PC (2013). CD8+ T cell-induced expression of tissue inhibitor of metalloproteinses-1 exacerbated osteoarthritis. Int J Mol Sci.

[16] Malfait AM, Little CB (2015). On the predictive utility of animal models of osteoarthritis. Arthritis Res Ther.

[17] Malfait AM, Little CB, McDougall JJ (2013). A commentary on modelling osteoarthritis pain in small animals. Osteoarthr Cartil.

[18] Jackson MT, Moradi B, Zaki S, Smith MM, McCracken S, Smith SM, Jackson CJ, Little CB (2014). Depletion of protease-activated receptor 2 but not protease-activated receptor 1 may confer protection against osteoarthritis in mice through extracartilaginous mechanisms. Arthritis Rheumatol.

[19] Cake M, Read R, Edwards S, Smith MM, Burkhardt D, Little C, Ghosh P (2008). Changes in gait after bilateral meniscectomy in sheep: effect of two hyaluronan preparations. J Orthop Sci.

[20] Smith MM, Cake MA, Ghosh P, Schiavinato A, Read RA, Little CB (2008). Significant synovial pathology in a meniscectomy model of osteoarthritis: modification by intra-articular hyaluronan therapy. Rheumatology (Oxford).

[21] Barry F, Murphy M (2013). Mesenchymal stem cells in joint disease and repair. Nat Rev Rheumatol.

[22] Jevotovsky DS, Alfonso AR, Einhorn TA, Chiu ES (2018). Osteoarthritis and stem cell therapy in humans: a systematic review. Osteoarthr Cartil.

[23] Schelbergen RF, van Dalen S, ter Huurne M, Roth J, Vogl T, Noel D, Jorgensen C, van den Berg WB, van de Loo FA, Blom AB (2014). Treatment efficacy of adipose-derived stem cells in experimental osteoarthritis is driven by high synovial activation and reflected by S100A8/A9 serum levels. Osteoarthr Cartil.

[24] Ter Huurne M, Schelbergen R, Blattes R, Blom A, de Munter W, Grevers LC, Jeanson J, Noel D, Casteilla L, Jorgensen C (2012). Antiinflammatory and chondroprotective effects of intraarticular injection of adipose-derived stem cells in experimental osteoarthritis. Arthritis Rheum.

[25] Shu CC, Jackson MT, Smith MM, Smith SM, Penm S, Lord MS, Whitelock JM, Little CB, Melrose J (2016). Ablation of perlecan domain 1 heparan sulfate reduces progressive cartilage degradation, synovitis, and osteophyte size in a preclinical model of posttraumatic osteoarthritis. Arthritis Rheumatol.

[26] Malfait AM, Ritchie J, Gil AS, Austin JS, Hartke J, Qin W, Tortorella MD, Mogil JS (2010). ADAMTS-5 deficient mice do not develop mechanical allodynia associated with osteoarthritis following medial meniscal destabilization. Osteoarthr Cartil.

[27] Ma HL, Blanchet TJ, Peluso D, Hopkins B, Morris EA, Glasson SS (2007). Osteoarthritis severity is sex dependent in a surgical mouse model. Osteoarthr Cartil.

[28] Nadri S, Soleimani M, Hosseni RH, Massumi M, Atashi A, Izadpanah R (2007). An efficient method for isolation of murine bone marrow mesenchymal stem cells. Int J Dev Biol.

[29] Shu C, Smith SM, Little CB, Melrose J (2016). Use of FGF-2 and FGF-18 to direct bone marrow stromal stem cells to chondrogenic and osteogenic lineages. Future science OA.

[30] Dixon WJ (1980). Efficient analysis of experimental observations. Annu Rev Pharmacol Toxicol.

[31] Tsang AS, Dart AJ, Biasutti SA, Jeffcott LB, Smith MM, Little CB (2019). Effects of tendon injury on uninjured regional tendons in the distal limb: an in-vivo study using an ovine tendinopathy model. PLoS One.

[32] McCurdy RD, McGrath JJ, Mackay-Sim A (2008). Validation of the comparative quantification method of real-time PCR analysis and a cautionary tale of housekeeping gene selection. Gene Ther Mol Biol.

[33] Bustin SA (2002). Quantification of mRNA using real-time reverse transcription PCR (RT-PCR): trends and problems. J Mol Endocrinol.

[34] Little CB, Barai A, Burkhardt D, Smith SM, Fosang AJ, Werb Z, Shah M, Thompson EW (2009). Matrix metalloproteinase 13-deficient mice are resistant to osteoarthritic cartilage erosion but not chondrocyte hypertrophy or osteophyte development. Arthritis Rheum.

[35] Muramatsu Y, Sasho T, Saito M, Yamaguchi S, Akagi R, Mukoyama S, Akatsu Y, Katsuragi J, Fukawa T, Endo J (2014). Preventive effects of hyaluronan from deterioration of gait parameters in surgically induced mice osteoarthritic knee model. Osteoarthr Cartil.

[36] Balazs EA (2004). Viscosupplementation for treatment of osteoarthritis: from initial discovery to current status and results. Surg Technol Int.

[37] Little CB, Meeker CT, Golub SB, Lawlor KE, Farmer PJ, Smith SM, Fosang AJ (2007). Blocking aggrecanase cleavage in the aggrecan interglobular domain abrogates cartilage erosion and promotes cartilage repair. J Clin Invest.

[38] Lohmander LS, Hellot S, Dreher D, Krantz EF, Kruger DS, Guermazi A, Eckstein F (2014). Intraarticular sprifermin (recombinant human fibroblast growth factor 18) in knee osteoarthritis: a randomized, double-blind, placebo-controlled trial. Arthritis Rheumatol.

[39] Stoker AM, Cook JL, Kuroki K, Fox DB (2006). Site-specific analysis of gene expression in early osteoarthritis using the Pond-Nuki model in dogs. J Orthop Surg Res.

[40] Young AA, Smith MM, Smith SM, Cake MA, Ghosh P, Read RA, Melrose J, Sonnabend DH, Roughley PJ, Little CB (2005). Regional assessment of articular cartilage gene expression and small proteoglycan metabolism in an animal model of osteoarthritis. Arthritis Res Ther.

[41] Harrell CR, Markovic BS, Fellabaum C, Arsenijevic A, Volarevic V (2019). Mesenchymal stem cell-based therapy of osteoarthritis: current knowledge and future perspectives. Biomed Pharmacother.

[42] Jayaram P, Ikpeama U, Rothenberg JB, Malanga GA (2018). Bone marrow-derived and adipose-derived mesenchymal stem cell therapy in primary knee osteoarthritis: a narrative review.

[43] Diekman BO, Wu CL, Louer CR, Furman BD, Huebner JL, Kraus VB, Olson SA, Guilak F (2013). Intra-articular delivery of purified mesenchymal stem cells from C57BL/6 or MRL/MpJ superhealer mice prevents posttraumatic arthritis. Cell Transplant.

[44] Sun Q, Nakata H, Yamamoto M, Kasugai S, Kuroda S (2019). Comparison of gingiva-derived and bone marrow mesenchymal stem cells for osteogenesis. J Cell Mol Med.

[45] Tang Y, Pan ZY, Zou Y, He Y, Yang PY, Tang QQ, Yin F (2017). A comparative assessment of adipose-derived stem cells from subcutaneous and visceral fat as a potential cell source for knee osteoarthritis treatment. J Cell Mol Med.

[46] Rostovskaya M, Anastassiadis K (2012). Differential expression of surface markers in mouse bone marrow mesenchymal stromal cell subpopulations with distinct lineage commitment. PLoS One.

[47] Bateman JF, Rowley L, Belluoccio D, Chan B, Bell K, Fosang AJ, Little CB (2013). Transcriptomics of wild-type mice and mice lacking ADAMTS-5 activity identifies genes involved in osteoarthritis initiation and cartilage destruction. Arthritis Rheum.

[48] Kung LHW, Ravi V, Rowley L, Angelucci C, Fosang AJ, Bell KM, Little CB, Bateman JF (2018). Cartilage microRNA dysregulation during the onset and progression of mouse osteoarthritis is independent of aggrecanolysis and overlaps with candidates from end-stage human disease. Arthritis Rheumat.

[49] Minten M.J.M., Blom A., Snijders G.F., Kloppenburg M., van den Hoogen F.H.J., den Broeder A.A., van der Kraan P.M., van den Ende C.H.M. (2019). Exploring longitudinal associations of histologically assessed inflammation with symptoms and radiographic damage in knee osteoarthritis: combined results of three prospective cohort studies. Osteoarthritis and Cartilage.

[50] Lin J, Wu G, Zhao Z, Huang Y, Chen J, Fu C, Ye J, Liu X (2018). Bioinformatics analysis to identify key genes and pathways influencing synovial inflammation in osteoarthritis. Mol Med Rep.

[51] Bali KK, Venkataramani V, Satagopam VP, Gupta P, Schneider R, Kuner R (2013). Transcriptional mechanisms underlying sensitization of peripheral sensory neurons by granulocyte-/granulocyte-macrophage colony stimulating factors. Mol Pain.

[52] Roberts S., Evans H., Wright K., van Niekerk L., Caterson B., Richardson J.B., Kumar K.H.S., Kuiper J.H. (2015). ADAMTS-4 activity in synovial fluid as a biomarker of inflammation and effusion. Osteoarthritis and Cartilage.

[53] Glasson S, Askew R, Sheppard B, Carito B, Blanchet T, Ma H, Flannery C, Kanki K, Wang E, Peluso D (2004). Characterization of and osteoarthritis susceptibility in ADAMTS-4-knockout mice. Arthritis Rheum.

[54] Majumdar MK, Askew R, Schelling S, Stedman N, Blanchet T, Hopkins B, Morris EA, Glasson SS (2007). Double-knockout of ADAMTS-4 and ADAMTS-5 in mice results in physiologically normal animals and prevents the progression of osteoarthritis. Arthritis Rheum.

[55] Glasson SS, Askew R, Sheppard B, Carito B, Blanchet T, Ma HL, Flannery CR, Peluso D, Kanki K, Yang Z (2005). Deletion of active ADAMTS5 prevents cartilage degradation in a murine model of osteoarthritis. Nature.

[56] Stanton H, Rogerson FM, East CJ, Golub SB, Lawlor KE, Meeker CT, Little CB, Last K, Farmer PJ, Campbell IK (2005). ADAMTS5 is the major aggrecanase in mouse cartilage in vivo and in vitro. Nature.

[57] Sorge RE, Mapplebeck JC, Rosen S, Beggs S, Taves S, Alexander JK, Martin LJ, Austin JS, Sotocinal SG, Chen D (2015). Different immune cells mediate mechanical pain hypersensitivity in male and female mice. Nat Neurosci.

[58] Baddack-Werncke U, Busch-Dienstfertig M, Gonzalez-Rodriguez S, Maddila SC, Grobe J, Lipp M, Stein C, Muller G (2017). Cytotoxic T cells modulate inflammation and endogenous opioid analgesia in chronic arthritis. J Neuroinflammation.

[59] Yang M, Peyret C, Shi XQ, Siron N, Jang JH, Wu S, Fournier S, Zhang J (2015). Evidence from human and animal studies: pathological roles of CD8(+) T cells in autoimmune peripheral neuropathies. Front Immunol.

[60] Klein-Wieringa IR, de Lange-Brokaar BJ, Yusuf E, Andersen SN, Kwekkeboom JC, Kroon HM, van Osch GJ, Zuurmond AM, Stojanovic-Susulic V, Nelissen RG (2016). Inflammatory cells in patients with endstage knee osteoarthritis: a comparison between the synovium and the infrapatellar fat pad. J Rheumatol.

[61] Martinez FO, Sica A, Mantovani A, Locati M (2008). Macrophage activation and polarization. Front Biosci.

[62] Sindrilaru A, Scharffetter-Kochanek K (2013). Disclosure of the culprits: macrophages-versatile regulators of wound healing. Adv Wound Care.

[63] Remst DF, Blaney Davidson EN, van der Kraan PM (2015). Unravelling osteoarthritis-related synovial fibrosis: a step closer to solving joint stiffness. Rheumatology (Oxford).

[64] Loeser RF, Olex AL, McNulty MA, Carlson CS, Callahan MF, Ferguson CM, Chou J, Leng X, Fetrow JS (2012). Microarray analysis reveals age-related differences in gene expression during the development of osteoarthritis in mice. Arthritis Rheum.

[65] Rowe MA, Harper LR, McNulty MA, Lau AG, Carlson CS, Leng L, Bucala RJ, Miller RA, Loeser RF (2017). Reduced osteoarthritis severity in aged mice with deletion of macrophage migration inhibitory factor. Arthritis Rheumatol.

