# Supplementary material for: The relationship between synovial inflammation, structural pathology, and pain in post-traumatic osteoarthritis: differential effect of stem cell and hyaluronan treatment
Source: Arthritis Res Ther. 2020 Feb 14;22:29. doi: 10.1186/s13075-020-2117-2 (PMC7023816; doi:10.1186/s13075-020-2117-2)
Supplement: Supplementary file 1 — Additional file 1: Table S1. Fluorescence activated cell sorting (FACS) analysis of the number (Mean ± SEM) of different synovial tissue inflammatory cell subtypes at 4, 8 and 12 weeks post-DMM (n = 5/treatment/time). Numbers highlighted in bold are significantly different (P<0.05) than for the same cell subtype in saline treated animals at that same time. Numbers marked with an * in weeks 8 and 12 are significantly different (P<0.05) than for the same cell subtype at 4 weeks in that treatment group. Numbers in italic text in week 12 are significantly different (P<0.05) than for the same cell subtype at 8 weeks in that treatment group. [file 13075_2020_2117_MOESM1_ESM.docx]

**Table S1:** Fluorescence activated cell sorting (FACS) analysis of the number (Mean ± SEM) of different synovial tissue inflammatory cell subtypes at 4, 8 and 12 weeks post-DMM (n = 5/treatment/time). Animals received intra-articular injection of Saline, Hymovis (Hym), MSCs, or MSCs followed by Hym, 2 and 4 weeks post-DMM. **Bold** = *P* < 0.05 compared with saline for that cell type at the same timepoint; * = *P* < 0.05 compared with week 4 for that cell type in the same treatment group; *Italic = P* < 0.05 compared with week 8 for that cell type in the same treatment group.

**A) Lymphocyte viable cell numbers**

| **4 weeks** | **Saline** | **Hym** | **MSC** | **M+H** |
| --- | --- | --- | --- | --- |
| CD3+ | 5428 ± 995 | **2266 ± 450** | **1708 ± 417** | **1447 ± 349** |
| CD3+/CD4+ | 2561 ± 560 | 1159 ± 348 | **718 ±197** | **446 ± 96** |
| CD3+/CD8+ | 368 ± 41 | **128 ± 21** | **125 ± 30** | **103 ± 28** |
| **8 weeks** |  |  |  |  |
| CD3+ | 278 ± 89 * | **1192 ± 368** | 765 ± 250 | 927 ± 345 |
| CD3+/CD4+ | 138 ± 49 * | **430 ± 200** | **195 ± 61 *** | **487 ± 300** |
| CD3+/CD8+ | 42 ± 31 * | **101 ± 26** | **103 ± 37** | **94 ± 18** |
| **12 weeks** |  |  |  |  |
| CD3+ | *2602 ± 964* | *2465 ± 407* | 1873 ± 1397 | 1185 ± 690 |
| CD3+/CD4+ | 976 ± 382 * | **876 ±183** | **612 ± 460** | **305 ± 209** |
| CD3+/CD8+ | 368 ± 41 | **128 ± 21** | **125 ± 30** | **103 ± 28** |

**B) Monocyte/macrophage viable cell numbers**

| **4 weeks** | **Saline** | **Hym** | **MSC** | **M+H** |
| --- | --- | --- | --- | --- |
| CD11b+ | 6162 ± 1112 | **1231 ± 331** | **2213 ± 428** | **1773 ± 407** |
| CD11b+ Ly6c+ | 1260 ± 559 | 310 ± 71 | 594 ± 135 | 689 ± 168 |
| CD11b+ F4/80+Ly6c+ | 533 ± 135 | 422 ± 77 | 372 ± 71 | 299 ± 50 |
| CD11b+ F4/80+ | 810 ± 159 | **227 ± 23** | **252 ± 71** | **235 ± 116** |
| **8 weeks** |  |  |  |  |
| CD11b+ | 324 ± 95 * | 372 ± 78 * | 347 ± 109 * | 282 ± 72 * |
| CD11b+ Ly6c+ | 167 ± 45 | 96 ± 26 * | 80 ± 31 * | 76 ± 21 * |
| CD11b+ F4/80+Ly6c+ | 89 ± 30 * | 90 ± 15 * | 117 ± 32 * | 139 ± 24 * |
| CD11b+ F4/80+ | 57 ± 24 * | 43 ± 18 * | 51 ± 21 * | 45 ± 7 |
| **12 weeks** |  |  |  |  |
| CD11b+ | 299 ± 40 * | 266 ± 63 * | 282 ± 158 * | 197 ± 74 * |
| CD11b+ Ly6c+ | 93 ± 18 | 96 ± 35 * | 91 ± 51 * | 47 ± 18 * |
| CD11b+ F4/80+Ly6c+ | 119 ± 29 * | 69 ± 35 * | 116 ± 48 * | 74 ± 35 * |
| CD11b+ F4/80+ | 66 ± 18 * | 38 ± 17 * | 43 ± 14 * | 37 ± 8 |

**C) M1/M2 Macrophage viable cell numbers**

| **4 weeks** | **Saline** | **Hym** | **MSC** | **M+H** |
| --- | --- | --- | --- | --- |
| F4/80+ | 3565 ± 434 | **1753 ± 301** | **1767 ± 385** | **1715 ± 212** |
| F4/80+ CD11c+ | 296 ± 99 | 165 ± 40 | 159 ± 39 | 151 ± 44 |
| F4/80+ CD206+ | 1615 ± 283 | 821 ± 275 | 777 ± 239 | **656 ± 99** |
| **8 weeks** |  |  |  |  |
| F4/80+ | 1049 ± 530 * | 1482 ± 272 | 1627 ± 251 | 932 ± 343 |
| F4/80+ CD11c+ | 140 ± 59 | 135 ± 49 | 190 ± 72 | 129 ± 66 |
| F4/80+ CD206+ | 314 ± 165 * | 547 ± 144 | 482 ± 82 | 347 ± 214 |
| **12 weeks** |  |  |  |  |
| F4/80+ | 2258 ± 710 | *4048 ± 1024* | 2496 ± 1297 | 1860 ± 690 |
| F4/80+ CD11c+ | 251 ± 71 | 474 ± 153 | 126 ± 34 | 271 ± 85 |
| F4/80+ CD206+ | 816 ± 312 | 1254 ± 313 | 945 ± 493 | 286 ± 54 * |

**D) M2a/M2c Macrophage viable cell numbers**

| **4 weeks** | **Saline** | **Hym** | **MSC** | **M+H** |
| --- | --- | --- | --- | --- |
| CD206+ | 8802 ± 680 | **4227 ± 1139** | **2758 ± 1092** | **2350 ± 469** |
| CD206+ CD301+ | 1025 ± 182 | **401 ± 139** | **323 ± 113** | **273 ± 44** |
| CD206+ CD150+ | 2276 ± 207 | **1239 ± 317** | **774 ± 323** | **558 ± 80** |
| **8 weeks** |  |  |  |  |
| CD206+ | 1002 ± 686 * | 2888 ± 1484 | 1809 ± 247 | 1769 ± 857 |
| CD206+ CD301+ | 113 ± 54 * | 176 ± 49 | 154 ± 56 | 93 ± 20 |
| CD206+ CD150+ | 418 ± 314 * | 877 ± 200 | 466 ± 157 | 399 ± 218 |
| **12 weeks** |  |  |  |  |
| CD206+ | 4299 ± 1631* | 4933 ± 1107 | 3749 ± 2114 | 1636 ± 569 |
| CD206+ CD301+ | 455 ± 179 | 622 ± 203 | 424 ± 227 | 161 ± 26 |
| CD206+ CD150+ | 1270 ± 462 | 1534 ± 371 | 1242 ± 710 | 661 ± 290 |
